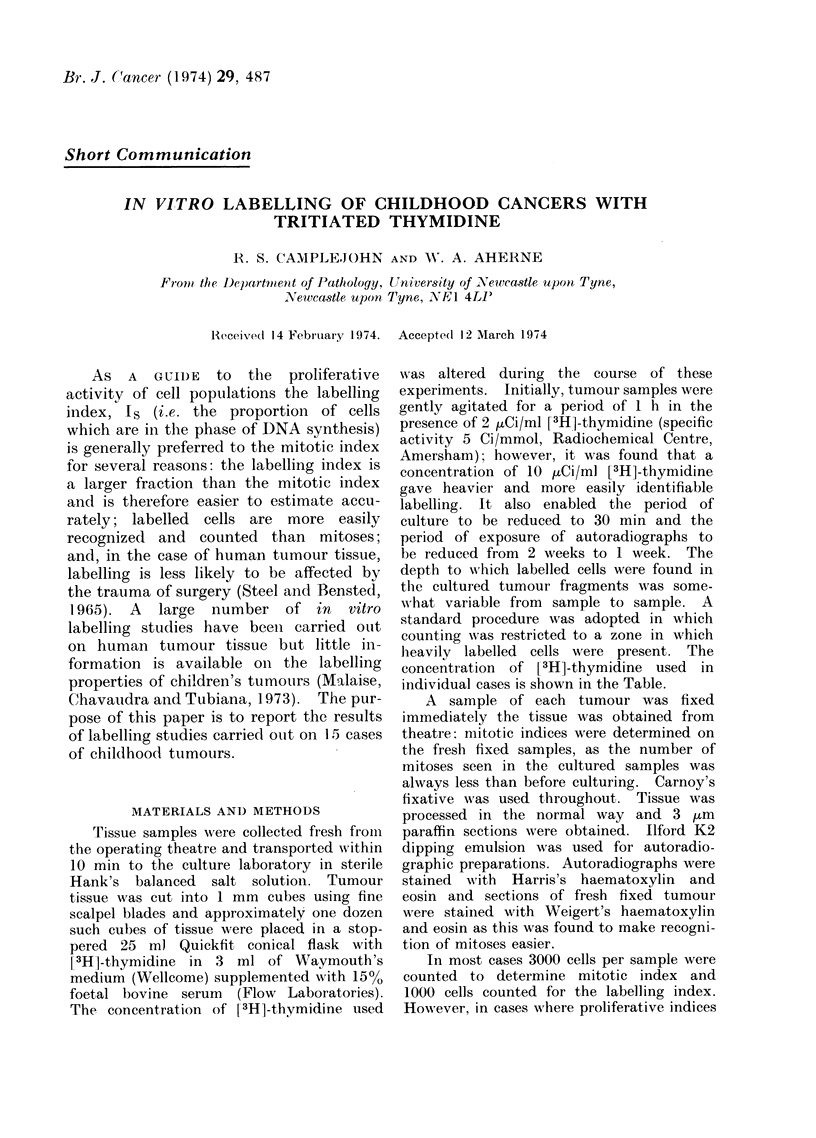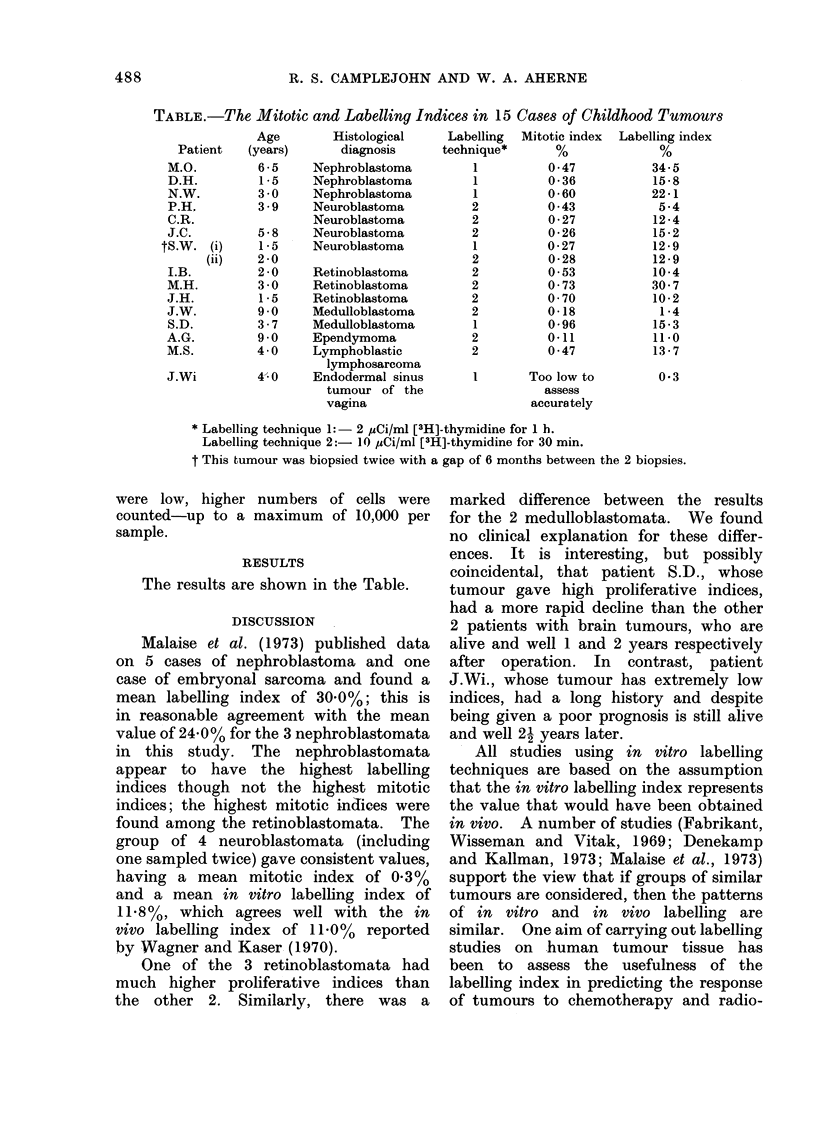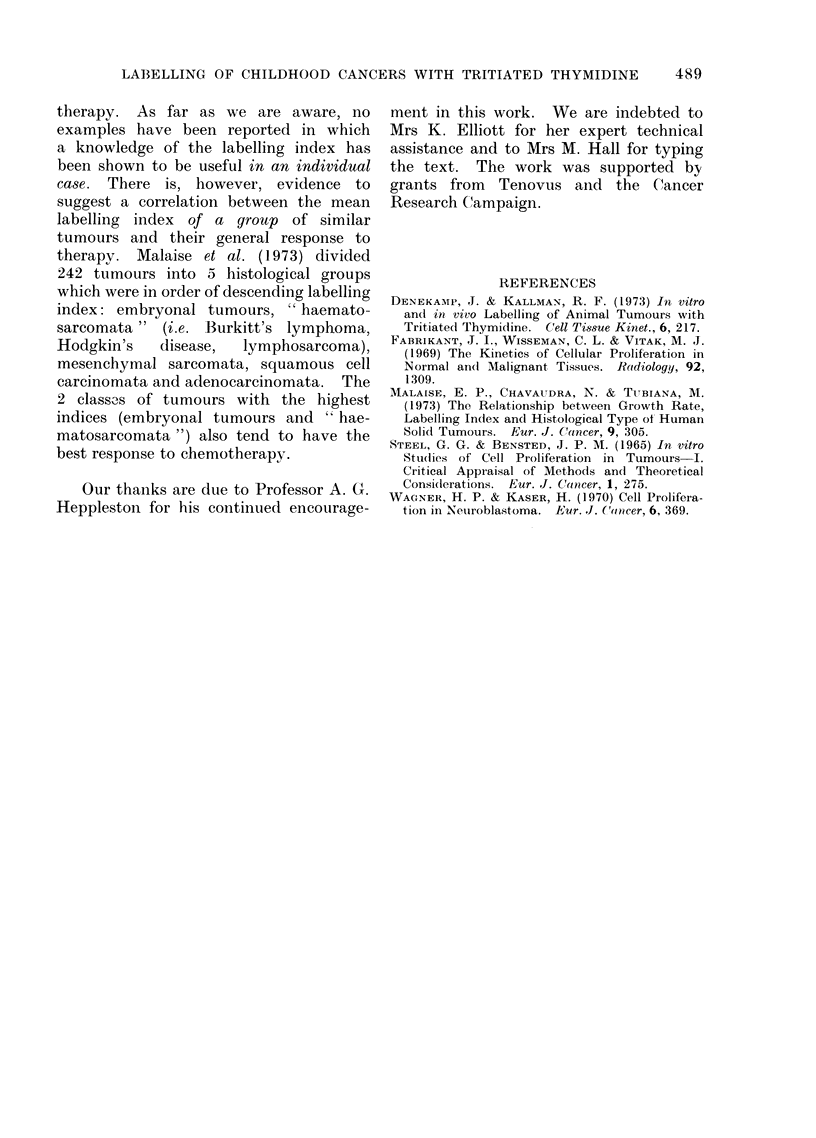# In vitro labelling of childhood cancers with tritiated thymidine.

**DOI:** 10.1038/bjc.1974.102

**Published:** 1974-06

**Authors:** R. S. Camplejohn, W. A. Aherne


*Br. J. Cancer* (1974) **29**, 487

***Short Communication***

### *IN VITRO* LABELLING OF CHILDHOOD CANCERS WITH TRITIATED THYMIDINE

R. S. CAMPLEJOHN AND W. A. AHERNE

*From the Department of Pathology, University of Newcastle upon Tyne, Newcastle upon Tyne, NE1 4LP*

Received 14 February 1974. Accepted 12 March 1974

As A GUIDE to the proliferative activity of cell populations the labelling index, $I_S$ (*i.e.* the proportion of cells which are in the phase of DNA synthesis) is generally preferred to the mitotic index for several reasons: the labelling index is a larger fraction than the mitotic index and is therefore easier to estimate accurately; labelled cells are more easily recognized and counted than mitoses; and, in the case of human tumour tissue, labelling is less likely to be affected by the trauma of surgery (Steel and Bensted, 1965). A large number of *in vitro* labelling studies have been carried out on human tumour tissue but little information is available on the labelling properties of children's tumours (Malaise, Chavaudra and Tubiana, 1973). The purpose of this paper is to report the results of labelling studies carried out on 15 cases of childhood tumours.

### MATERIALS AND METHODS

Tissue samples were collected fresh from the operating theatre and transported within 10 min to the culture laboratory in sterile Hank's balanced salt solution. Tumour tissue was cut into 1 mm cubes using fine scalpel blades and approximately one dozen such cubes of tissue were placed in a stoppered 25 ml Quickfit conical flask with [$^3$H]-thymidine in 3 ml of Waymouth's medium (Wellcome) supplemented with 15% foetal bovine serum (Flow Laboratories). The concentration of [$^3$H]-thymidine used was altered during the course of these experiments. Initially, tumour samples were gently agitated for a period of 1 h in the presence of 2 $\mu$Ci/ml [$^3$H]-thymidine (specific activity 5 Ci/mmol, Radiochemical Centre, Amersham); however, it was found that a concentration of 10 $\mu$Ci/ml [$^3$H]-thymidine gave heavier and more easily identifiable labelling. It also enabled the period of culture to be reduced to 30 min and the period of exposure of autoradiographs to be reduced from 2 weeks to 1 week. The depth to which labelled cells were found in the cultured tumour fragments was somewhat variable from sample to sample. A standard procedure was adopted in which counting was restricted to a zone in which heavily labelled cells were present. The concentration of [$^3$H]-thymidine used in individual cases is shown in the Table.

A sample of each tumour was fixed immediately the tissue was obtained from theatre: mitotic indices were determined on the fresh fixed samples, as the number of mitoses seen in the cultured samples was always less than before culturing. Carnoy's fixative was used throughout. Tissue was processed in the normal way and 3 $\mu$m paraffin sections were obtained. Ilford K2 dipping emulsion was used for autoradiographic preparations. Autoradiographs were stained with Harris's haematoxylin and eosin and sections of fresh fixed tumour were stained with Weigert's haematoxylin and eosin as this was found to make recognition of mitoses easier.

In most cases 3000 cells per sample were counted to determine mitotic index and 1000 cells counted for the labelling index. However, in cases where proliferative indices

TABLE.—*The Mitotic and Labelling Indices in* 15 *Cases of Childhood Tumours*

| Patient | Age (years) | Histological diagnosis | Labelling technique* | Mitotic index % | Labelling index % |
|---|---|---|---|---|---|
| M.O. | 6·5 | Nephroblastoma | 1 | 0·47 | 34·5 |
| D.H. | 1·5 | Nephroblastoma | 1 | 0·36 | 15·8 |
| N.W. | 3·0 | Nephroblastoma | 1 | 0·60 | 22·1 |
| P.H. | 3·9 | Neuroblastoma | 2 | 0·43 | 5·4 |
| C.R. | | Neuroblastoma | 2 | 0·27 | 12·4 |
| J.C. | 5·8 | Neuroblastoma | 2 | 0·26 | 15·2 |
| †S.W. (i) | 1·5 | Neuroblastoma | 1 | 0·27 | 12·9 |
| (ii) | 2·0 | | 2 | 0·28 | 12·9 |
| I.B. | 2·0 | Retinoblastoma | 2 | 0·53 | 10·4 |
| M.H. | 3·0 | Retinoblastoma | 2 | 0·73 | 30·7 |
| J.H. | 1·5 | Retinoblastoma | 2 | 0·70 | 10·2 |
| J.W. | 9·0 | Medulloblastoma | 2 | 0·18 | 1·4 |
| S.D. | 3·7 | Medulloblastoma | 1 | 0·96 | 15·3 |
| A.G. | 9·0 | Ependymoma | 2 | 0·11 | 11·0 |
| M.S. | 4·0 | Lymphoblastic lymphosarcoma | 2 | 0·47 | 13·7 |
| J.Wi | 4·0 | Endodermal sinus tumour of the vagina | 1 | Too low to assess accurately | 0·3 |

* Labelling technique 1:— 2 μCi/ml [$^3$H]-thymidine for 1 h.

Labelling technique 2:— 10 μCi/ml [$^3$H]-thymidine for 30 min.

† This tumour was biopsied twice with a gap of 6 months between the 2 biopsies.

were low, higher numbers of cells were counted—up to a maximum of 10,000 per sample.

### RESULTS

The results are shown in the Table.

### DISCUSSION

Malaise *et al.* (1973) published data on 5 cases of nephroblastoma and one case of embryonal sarcoma and found a mean labelling index of 30·0%; this is in reasonable agreement with the mean value of 24·0% for the 3 nephroblastomata in this study. The nephroblastomata appear to have the highest labelling indices though not the highest mitotic indices; the highest mitotic indices were found among the retinoblastomata. The group of 4 neuroblastomata (including one sampled twice) gave consistent values, having a mean mitotic index of 0·3% and a mean *in vitro* labelling index of 11·8%, which agrees well with the *in vivo* labelling index of 11·0% reported by Wagner and Kaser (1970).

One of the 3 retinoblastomata had much higher proliferative indices than the other 2. Similarly, there was a marked difference between the results for the 2 medulloblastomata. We found no clinical explanation for these differences. It is interesting, but possibly coincidental, that patient S.D., whose tumour gave high proliferative indices, had a more rapid decline than the other 2 patients with brain tumours, who are alive and well 1 and 2 years respectively after operation. In contrast, patient J.Wi., whose tumour has extremely low indices, had a long history and despite being given a poor prognosis is still alive and well 2½ years later.

All studies using *in vitro* labelling techniques are based on the assumption that the *in vitro* labelling index represents the value that would have been obtained *in vivo*. A number of studies (Fabrikant, Wisseman and Vitak, 1969; Denekamp and Kallman, 1973; Malaise *et al.*, 1973) support the view that if groups of similar tumours are considered, then the patterns of *in vitro* and *in vivo* labelling are similar. One aim of carrying out labelling studies on human tumour tissue has been to assess the usefulness of the labelling index in predicting the response of tumours to chemotherapy and radiotherapy. As far as we are aware, no examples have been reported in which a knowledge of the labelling index has been shown to be useful *in an individual case*. There is, however, evidence to suggest a correlation between the mean labelling index *of a group* of similar tumours and their general response to therapy. Malaise *et al.* (1973) divided 242 tumours into 5 histological groups which were in order of descending labelling index: embryonal tumours, "haematosarcomata" (*i.e.* Burkitt's lymphoma, Hodgkin's disease, lymphosarcoma), mesenchymal sarcomata, squamous cell carcinomata and adenocarcinomata. The 2 classes of tumours with the highest indices (embryonal tumours and "haematosarcomata") also tend to have the best response to chemotherapy.

Our thanks are due to Professor A. G. Heppleston for his continued encouragement in this work. We are indebted to Mrs K. Elliott for her expert technical assistance and to Mrs M. Hall for typing the text. The work was supported by grants from Tenovus and the Cancer Research Campaign.

### REFERENCES

DENEKAMP, J. & KALLMAN, R. F. (1973) *In vitro* and *in vivo* Labelling of Animal Tumours with Tritiated Thymidine. *Cell Tissue Kinet.*, **6**, 217.

FABRIKANT, J. I., WISSEMAN, C. L. & VITAK, M. J. (1969) The Kinetics of Cellular Proliferation in Normal and Malignant Tissues. *Radiology*, **92**, 1309.

MALAISE, E. P., CHAVAUDRA, N. & TUBIANA, M. (1973) The Relationship between Growth Rate, Labelling Index and Histological Type of Human Solid Tumours. *Eur. J. Cancer*, **9**, 305.

STEEL, G. G. & BENSTED, J. P. M. (1965) *In vitro* Studies of Cell Proliferation in Tumours—I. Critical Appraisal of Methods and Theoretical Considerations. *Eur. J. Cancer*, **1**, 275.

WAGNER, H. P. & KASER, H. (1970) Cell Proliferation in Neuroblastoma. *Eur. J. Cancer*, **6**, 369.